# Association Between ER/PR-Positive Breast Tumors and Digestive Cancers

**DOI:** 10.3390/diagnostics16132052

**Published:** 2026-06-30

**Authors:** Anca Andreea Nica, Traian Pătrașcu, Vlad Denis Constantin, Ruxandra Viorica Stănculescu, Bogdan Socea, Alexandru Constantin Carâp, Andreea Dragon

**Affiliations:** 1 Department of General Surgery, Sfântul Pantelimon Emergency Clinical Hospital, Sos Pantelimon nr 340-342, 021659 Bucharest, Romania; 2 The Faculty of General Medicine, Carol Davila University of Medicine and Pharmacy, 050474 Bucharest, Romania; 3 Department of General Surgery, Clinical Hospital Dr. Ion Cantacuzino, 021659 Bucharest, Romania

**Keywords:** breast cancer, ER/PR-positive, digestive tumors

## Abstract

**Background/Objectives:** Breast cancer is the most commonly diagnosed malignancy among women, with hormone receptor-positive tumors representing the majority of cases. Increasing survival rates have shifted attention toward long-term complications, including the risk of secondary malignancies. Emerging evidence suggests a potential association between breast cancer and gastrointestinal (GI) neoplasia. This study aimed to evaluate the role of colonoscopic and upper gastrointestinal endoscopic monitoring in patients with ER/PR-positive breast cancer and to assess its potential value in the early detection of digestive lesions. **Methods:** We conducted a prospective observational study including 186 female patients with histologically confirmed ER/PR-positive breast cancer. A total of 95 patients underwent colonoscopy, and 91 patients underwent upper gastrointestinal endoscopy. Clinical, demographic, and risk factor data were collected. A structured questionnaire was used to assess gastrointestinal symptoms. Endoscopic findings, lesion characteristics, and histopathological results were recorded. Bowel preparation quality was assessed using the Boston Bowel Preparation Scale. **Results:** Colonoscopy identified polyps and other lesions, with the majority located in the rectum and descending colon. A total of 12 biopsies were performed, revealing 1 malignant lesion, 2 borderline lesions, and the remainder benign. Upper gastrointestinal endoscopy showed gastritis as the most frequent finding, followed by gastric ulcers and polyps, while most patients had normal endoscopic results. Overall, 72% of patients presented at least one risk factor for digestive malignancy. Following treatment, most patients reported improvement in gastrointestinal symptoms. **Conclusions:** Patients with ER/PR-positive breast cancer may present a higher prevalence of gastrointestinal lesions, potentially related to shared risk factors and the systemic effects of endocrine therapy. Targeted, symptom-oriented endoscopic evaluation may facilitate early detection of premalignant and malignant digestive conditions. A multidisciplinary, risk-adapted surveillance approach should be considered to improve patient outcomes. Further large-scale studies are required to establish evidence-based screening strategies in this population.

## 1. Introduction

Breast cancer remains the most frequently diagnosed malignancy among women worldwide, with estrogen receptor (ER)- and progesterone receptor (PR)-positive tumors accounting for the majority of cases [1,2]. Advances in early detection and targeted therapies have significantly improved survival outcomes, resulting in a growing population of long-term breast cancer survivors. As survival increases, attention has shifted toward the identification and management of long-term complications, including the risk of second primary malignancies [3,4].

Emerging evidence suggests a potential association between breast cancer and gastrointestinal (GI) neoplasia, particularly colorectal cancer [5]. Shared risk factors such as obesity, hormonal influences, genetic susceptibility, and lifestyle-related factors may contribute to this association. Estrogen and progesterone play complex roles in gastrointestinal carcinogenesis, influencing cellular proliferation, inflammation, and tumor microenvironment dynamics. Consequently, patients with ER/PR-positive breast tumors may represent a distinct subgroup with a modified risk profile for digestive tract malignancies [6,7].

Recent studies have reported higher rates of colorectal adenomas and colorectal cancer among breast cancer survivors compared with the general population, with some data suggesting earlier onset of neoplastic lesions [8]. However, current screening recommendations for digestive cancers in breast cancer patients remain largely age-based and do not account for tumor hormonal status. The potential predictive value of ER/PR positivity for gastrointestinal neoplasia has not been sufficiently explored [9].

Colonoscopic and upper endoscopic surveillance are well-established tools for the early detection of premalignant and malignant digestive lesions. Identifying high-risk subgroups that may benefit from earlier or more intensive endoscopic monitoring is essential for optimizing cancer prevention strategies [10]. Evaluating whether ER/PR-positive breast cancer constitutes a predictive factor for digestive cancer could have important clinical implications, enabling personalized surveillance protocols and improving long-term outcomes [11,12].

Aromatase inhibitors [13] were introduced into clinical practice in the late 20th century as a major advancement in the endocrine treatment of hormone receptor-positive breast cancer. By inhibiting the aromatase enzyme responsible for the peripheral conversion of androgens into estrogens, these agents significantly reduce circulating estrogen levels in postmenopausal women. Early clinical trials demonstrated superior efficacy of third-generation aromatase inhibitors, such as anastrozole, letrozole, and exemestane, compared with tamoxifen in reducing recurrence rates and improving disease-free survival. As a result, AIs have become a cornerstone of adjuvant therapy and are widely used in both early-stage and advanced breast cancer.

Despite their well-established oncologic benefits, long-term use of aromatase inhibitors has been associated with a range of systemic effects, including metabolic changes, bone density loss, and cardiovascular risk. Increasing attention has therefore been directed toward understanding their broader impact on non-target tissues, including the gastrointestinal tract. Given the role of estrogen in maintaining mucosal integrity and modulating inflammatory responses, prolonged estrogen deprivation may have implications for gastrointestinal physiology and potentially contribute to the development of digestive tract pathology.

Aromatase inhibitors (AIs) are pillars of adjuvant endocrine therapy in postmenopausal patients with estrogen receptor-positive breast cancer owing to their ability to markedly suppress peripheral estrogen synthesis and reduce disease recurrence. However, despite improvements in disease-free survival, several large randomized trials and meta-analyses have demonstrated that prolonged use of AIs carries specific toxicity dealings that may influence overall treatment outcomes [5]. Notably, the systematic review by Amir et al. found that extended AI therapy was associated with increased odds of cardiovascular disease and bone fractures, while reducing the risks of venous thrombosis and endometrial carcinoma compared with tamoxifen, suggesting a complex risk–benefit profile for long-term AI use [5]. These adverse effects underscore the importance of vigilant monitoring in breast cancer survivors, as endocrine therapy can impact comorbid organ systems beyond the primary tumor [14]. Although epidemiological evidence does not consistently support a direct association between aromatase inhibitor therapy and gastrointestinal malignancies, including colorectal cancer, the systemic endocrine and metabolic perturbations induced by prolonged estrogen suppression may have implications for digestive tract carcinogenesis, thereby underscoring the necessity for vigilant and targeted endoscopic surveillance in this patient population.

This article aims to review and analyze the role of colonoscopic and endoscopic monitoring in patients with ER/PR-positive breast tumors, with a focus on their potential predictive value for digestive cancer development. By clarifying this association, we seek to contribute to evidence-based recommendations for tailored gastrointestinal surveillance in this growing patient population.

## 2. Materials and Methods

This study was designed as a prospective observational investigation conducted in the Surgery Ward of Saint Pantelimon Emergency Clinical Hospital, Bucharest. Patient enrollment began in November 2019 and continued through December 2025. All eligible patients admitted to the surgical ward during the study period were prospectively followed according to predefined inclusion and exclusion criteria, totaling a number of 186 patients.

Clinical, demographic, and procedural data were collected in real time using standardized data collection forms. Patients were managed according to institutional protocols and the treating surgeon’s clinical judgment; no interventions were assigned or modified as part of the study. Outcomes were recorded during hospitalization and, when applicable, during scheduled postoperative follow-up.

The observational nature of the study ensured that routine clinical practice was not altered.

**Study Population**—We included female patients with a confirmed diagnosis of breast cancer and available immunohistochemistry (IHC) showing ER- and PR-positive tumor receptors and HER −/+. Patients underwent colonoscopic or endoscopic evaluation, regardless of whether they received endocrine therapy.

In order to select the appropriate patients, we described and used the inclusion criteria: female patients, histologically confirmed breast cancer, ER- and PR-positive tumor receptors on IHC, and no previous colorectal imaging. The exclusion criteria were applied to filter out those who were not suitable for the interest of this study: inadequate bowel preparation, and diagnosis of inflammatory bowel disease (IBD).

**Data Collection**—The following variables were recorded: demographic data; family history of colorectal cancer; risk factors (body mass index—BMI, smoking status, nulliparity); endocrine therapy status (yes/no); colonoscopy findings; and endoscopic findings. The results of colonoscopies were analyzed with regard to: presence of polyps/polyp size; presence of benign lesions (diverticulae, colitis); anatomical localization; endoscopic removal (yes/no); and histological characteristics.

Endoscopic findings were systematically analyzed, including the presence of lesions, ulcer size and localization, the presence of gastric or esophageal polyps, and signs of gastritis.

## 3. Results

A total of 95 patients with a current or previous diagnosis of ER/PR-positive breast cancer who had undergone neoadjuvant therapy/had breast removal surgery or are taking hormones after surgery were included in the structured surveillance program. Colonoscopy was performed in 95 patients, while 91 patients underwent upper gastrointestinal endoscopy. Endoscopic evaluation was conducted as part of a predefined monitoring protocol designed to assess potential gastrointestinal pathologies in this high-risk oncologic population. Although the surveillance strategy was systematic, procedure selection was further guided by the presence of digestive symptoms reported at clinical evaluation. Lower gastrointestinal complaints, including altered bowel habits, constipation, or loose stools, reinforced the indication for colonoscopy, whereas upper gastrointestinal symptoms such as nausea, anorexia, epigastric discomfort, or unintentional weight loss supported the indication for upper endoscopy. Patients were assigned to either upper or lower endoscopy based on their predominant clinical presentation.

A structured questionnaire was administered to all patients prior to endoscopic evaluation (Table 1). The symptom assessment questionnaire was established through interdisciplinary collaboration with oncologists and was subsequently polished during routine clinical visits, incorporating patient-reported symptoms to ensure its relevance and value to this specific population.

While gastrointestinal endoscopic screening is not routinely recommended in patients with breast cancer, oncological clinical practice frequently highlights the occurrence of digestive symptoms during systemic therapy.

Patients were then informed about the clinical study that we were conducting and they agreed to complete the form; after that, we planned a visit for either colonoscopy or endoscopy.

To minimize potential confounding, patients were instructed to report symptoms based on their condition prior to the initiation of endocrine therapy (aromatase inhibitors) or, when applicable, prior to the diagnosis of breast cancer. This approach aimed to better reflect baseline gastrointestinal symptomatology.

This allowed for a more accurate differentiation between pre-existing gastrointestinal symptoms and those potentially induced by oncologic treatment.

**Figure 1:**
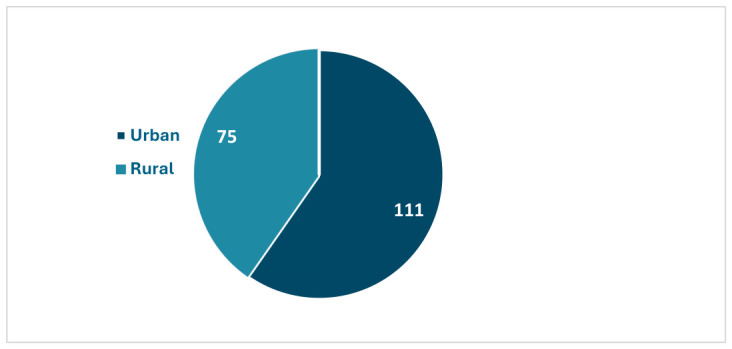
Demography: patients from urban residence were associated with greater accessibility to the hospital setting, reflecting shorter travel distances and easier access to healthcare facilities.

As shown in Figure 1, patients from urban residence were associated with greater accessibility to the hospital setting, reflecting shorter travel distances and easier access to healthcare facilities.

**Figure 2:**
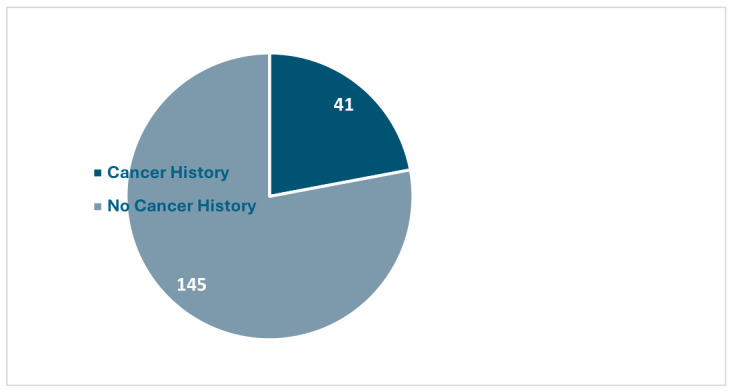
Family History of Malignancy: in this cohort of patients with breast cancer, the majority reported no family history of malignancy.

In this cohort of patients with breast cancer, the majority reported no family history of malignancy (Figure 2).

**Figure 3:**
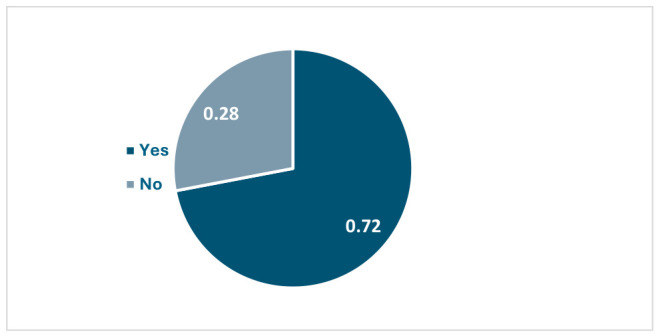
Risk factors: The majority of patients in our cohort (72%) presented at least one established risk factor for digestive malignancies.

The majority of patients in our cohort (72%) presented at least one established risk factor for digestive malignancies (Figure 3), including elevated body mass index, smoking, diabetes mellitus, alcohol consumption, and/or a positive family history. These findings are consistent with existing evidence identifying metabolic and lifestyle-related factors as significant contributors to gastrointestinal carcinogenesis. Obesity and diabetes, in particular, have been associated with chronic low-grade inflammation and insulin resistance, which may promote neoplastic transformation in the colorectal mucosa [14,15]. Similarly, smoking and alcohol consumption are well-recognized risk factors for colorectal cancer, exerting carcinogenic effects through both direct mucosal exposure and systemic mechanisms [16,17]. In addition, a positive family history has been shown to significantly increase the risk of colorectal cancer, likely reflecting both genetic predisposition and shared environmental factors [18]. The high prevalence of these risk factors in our study population may partially explain the frequency of detected lesions and suggests a cumulative effect when combined with hormonal and metabolic alterations associated with breast cancer and its treatment.

**Figure 4:**
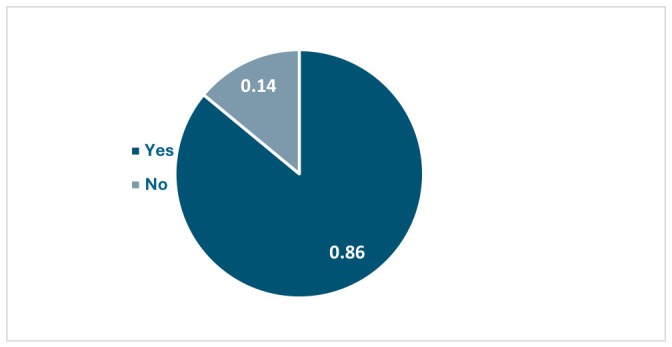
Endocrine therapy: several studies published in the last 10 years say that using Tamoxifen or aromatase inhibitors can influence secondary tumors in the digestive tract primarily through metastasis.

One important feature in the study was to enroll patients who received endocrine therapy. However, during follow-up some of them refused to continue primarily due to severe side effects that severely impact quality of life, including joint pain (arthralgia), severe hot flashes, fatigue, and depression. The long, 5–10 year, daily commitment is also daunting, with roughly 20–50% of patients failing to complete treatment. We had this in mind considering that several studies published in the last 10 years say that using Tamoxifen or aromatase inhibitors can influence secondary tumors in the digestive tract primarily through metastasis (Figure 4).

Bowel preparation was performed using polyethylene glycol-based solutions, with patients receiving clear instructions regarding dietary restrictions and timing of administration. Some patients completed the preparation while hospitalized under medical supervision, whereas others performed it at home. The quality of bowel cleansing was assessed during colonoscopy using the Boston Bowel Preparation Scale (BBPS). Overall, more than 70% of patients achieved adequate bowel preparation, while approximately 20% had suboptimal preparation. In 10 cases, patients were unable or unwilling to complete the preparation protocol. Poor preparation was more frequently observed among those who carried out the procedure at home. All colonoscopic examinations were performed using a flexible video endoscope (Olympus Evis Extra III CV-190 PLUS, Hamburg Germany), allowing for satisfactory visualization of the colonic mucosa in most cases.

**Figure 5:**
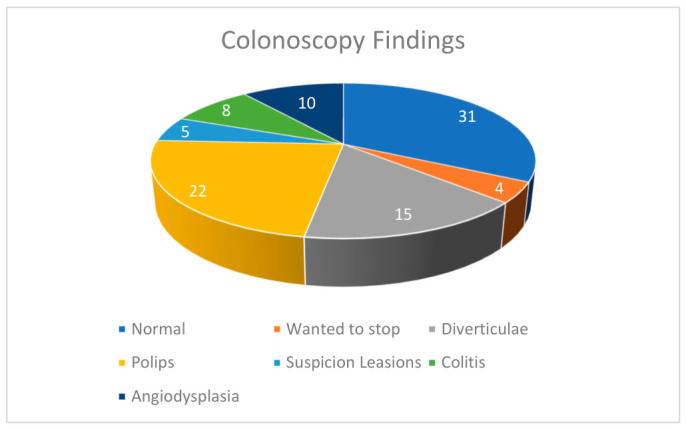
Colonoscopy findings.

From a total number of 95 patients that underwent colonoscopy, we biopsied five suspicious lesions and seven polyps. From that total number of 12 biopsies, 1 came back malignant, 2 borderline and the rest were benign (Figure 5).

**Figure 6:**
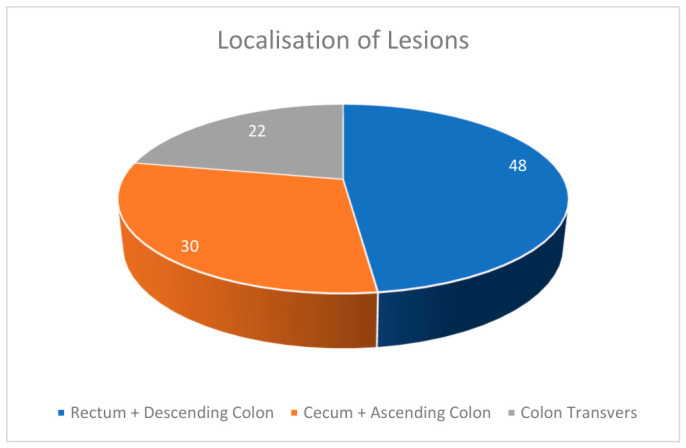
Localization of leasions.

We arranged all the information about the lesions we found in separate tables indicating the size of the lesion, localization, Paris classification for polyps and histopathological diagnosis. The majority were located in the rectum and descending colon, as shown in Figure 6.

The distribution of colorectal lesions in our cohort demonstrated a predominance in the rectum and descending colon, followed by the cecum and ascending colon, with fewer lesions identified in the transverse colon (Table 2). This pattern is consistent with previously reported data indicating that distal colorectal segments, particularly the rectosigmoid region, represent the most common sites for polyp detection in average-risk populations undergoing colonoscopic screening. The higher prevalence of lesions in the distal colon may be partially explained by differences in luminal exposure to carcinogenic factors, variations in mucosal biology, and longer transit time, which may promote neoplastic transformation. However, the presence of a substantial proportion of lesions in the proximal colon in our study also aligns with emerging evidence emphasizing the importance of complete colonoscopic evaluation, as right-sided lesions may be more frequently missed and can exhibit distinct molecular characteristics. These findings reinforce the need for thorough examination of the entire colon in patients with breast cancer, particularly in the context of shared risk factors and potential systemic influences related to endocrine therapy.

### Lesion Characteristics Table

The patient that had a positive rectal adenocarcinoma was also undergoing treatment for a breast carcinoma NST G1; she later went and had neoadjuvant treatment (Tamoxifen + Zoladex), radio and chemotherapy (Oxaliplatin) and continued with hormonal therapy.

Afterwards, she underwent a combined surgical procedure, including total mastectomy with axillary lymph node dissection and concurrent excision of the rectal tumor with colostomy.

**Figure 7:**
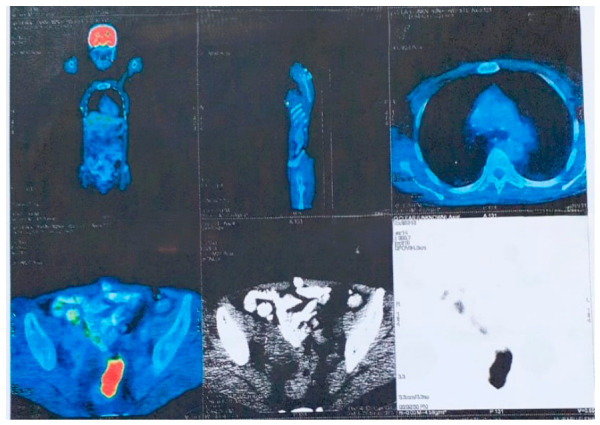
Patient nr 66—Pet CT showing metabolic activity in the rectum.

In this study, 91 patients underwent diagnostic upper gastrointestinal endoscopy. Endoscopic findings were categorized as gastric polyps, gastritis, small intestine ulcers, hemangioma, gastric ulcers, and adenocarcinoma. Gastritis was the most commonly identified pathology, followed by gastric ulcers and gastric polyps. Malignant pathology (adenocarcinoma) was detected in a small proportion of patients. The rest of the 70 patients had a normal endoscopy (Figure 7).

Upper gastrointestinal endoscopy was performed using a flexible video endoscope in accordance with standard clinical practice. All patients were evaluated after appropriate fasting (minimum 6–8 h), and the procedure was carried out under local pharyngeal anesthesia, with or without conscious sedation depending on patient tolerance and clinical indication.

**Figure 8:**
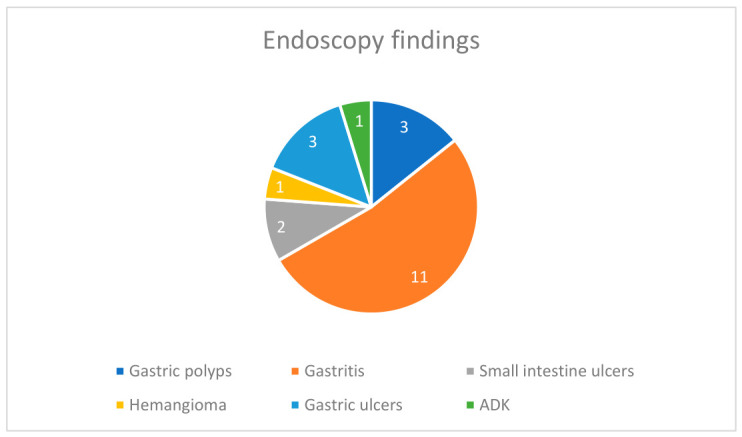
Endoscopy findings.

A systematic examination of the upper gastrointestinal tract was performed, including the esophagus, stomach, and duodenum (Figure 8). Careful inspection of the gastric mucosa was conducted, with particular attention to areas of erythema, erosion, ulceration, or polypoid lesions. When indicated, targeted biopsies were obtained from suspicious lesions, as well as from areas suggestive of gastritis, in accordance with standard biopsy protocols.

The presence of pathological findings such as gastritis, gastric ulcers, polyps, or other lesions was recorded (Figure 8). In selected cases, biopsies were also obtained for the detection of *Helicobacter pylori* infection. All procedures were well tolerated, and no major complications were observed.

Although gastrointestinal endoscopic screening is not routinely performed in patients with breast cancer, clinical observations indicate that digestive symptoms are frequently reported. Furthermore, the presence of aromatase activity within gastric parietal cells supports a potential biological mechanism through which estrogen deprivation may impair mucosal integrity and contribute to gastritis-like changes. These findings highlight the need for systematic endoscopic evaluation in symptomatic patients receiving endocrine therapy.

Immunotherapy for breast cancer (BC) has also made significant progress in recent years with the approval of various ICIs, especially in early-stage and metastatic TNBC [19,20,21]. However, in addition to the remarkable efficacy, immune-related adverse events (irAEs) have been observed in 30–60% of patients treated with ICIs, involving almost any organ of the body, including the skin, gastrointestinal (GI) tract, liver, thyroid, and heart [22].

A 64-year-old female patient, with a significant smoking history, was diagnosed two years prior with invasive lobular breast carcinoma. At that time, she underwent breast-conserving surgery by personal choice. Although axillary lymph node involvement was identified, the patient declined further surgical management, including axillary lymph node dissection and radical mastectomy.

**Figure 9:**
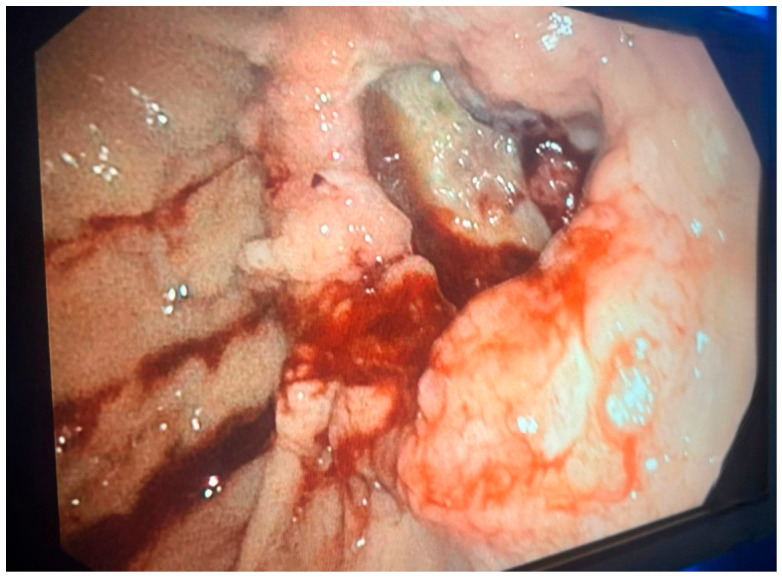
Patient nr 44 from the upper endoscopy lot.

She subsequently presented to our emergency department with severe anemia (hemoglobin 7.1 g/dL), hematemesis, and melena. Upper gastrointestinal endoscopy revealed a pseudotumoral lesion located on the lesser curvature of the stomach (as seen in Figure 9). Targeted biopsies were obtained, and histopathological examination confirmed the presence of gastric adenocarcinoma.

This case highlights the potential for synchronous or metachronous gastrointestinal malignancies in patients with breast cancer and underscores the importance of prompt endoscopic evaluation in the presence of alarm symptoms.

Therapeutic management was individualized based on endoscopic and histopathological findings. Acid suppression therapy with proton pump inhibitors was the foundation of treatment for gastritis and peptic ulcer disease. Targeted eradication therapy was administered in patients with confirmed Helicobacter pylori infection. Complementary treatment included antacids, mucosal protective agents, and prokinetics, depending on symptoms. Antispasmodics were used in selected patients with functional gastrointestinal symptoms. General dietary and lifestyle modifications were recommended in all cases.

Following treatment, most patients experienced an improvement in gastrointestinal symptoms, including a reduction in epigastric pain, nausea, and altered bowel movements. Symptom relief was observed particularly in patients receiving acid-suppressive therapy and targeted eradication of Helicobacter pylori infection. Although the degree of improvement varied among individuals, the overall trend suggests a positive clinical response to the instituted therapeutic measures.

## 4. Discussion

This prospective study provides insight into colorectal polyp detection and gastric premalignant lesions in patients with ER/PR-positive breast cancer. Shared hormonal and lifestyle-related risk factors may contribute to the observed findings. Our results are consistent with emerging evidence indicating an increased risk of gastrointestinal malignancies in breast cancer survivors, particularly colorectal cancer [19].

In addition, endocrine therapies such as aromatase inhibitors are associated with systemic effects that extend beyond breast tissue [22], and may influence gastrointestinal physiology.

From a pathophysiological perspective, the presence of aromatase activity within gastric mucosal cells supports a potential role of estrogen in maintaining mucosal integrity [23]. Consequently, estrogen deprivation induced by endocrine therapy may predispose patients to inflammatory or premalignant changes within the digestive tract. Furthermore, recent reports have demonstrated that cancer therapies, including immune checkpoint inhibitors, may induce inflammatory gastric lesions detectable on endoscopy [18]. Experimental data also suggest that hormonal modulation may directly affect gastric epithelial cells [24].

These findings underscore the potential value of targeted endoscopic evaluation in symptomatic patients with breast cancer. However, this study has limitations, including the relatively small sample size and the absence of a control group. Further large-scale studies are required to clarify the relationship between breast cancer, its treatment, and gastrointestinal pathology.

From a clinical perspective, these findings highlight the importance of not overlooking gastrointestinal symptoms in patients undergoing treatment for breast cancer. Symptoms such as dyspepsia, altered bowel habits, or weight loss are often attributed to treatment-related side effects; however, our results suggest that these manifestations may reflect underlying organic pathology detectable through endoscopic evaluation. Early identification of such lesions may allow timely intervention and potentially reduce the risk of progression to malignancy.

An important consideration raised by our study is whether gastrointestinal evaluation in this population should remain strictly symptom-driven or whether selected patients may benefit from a more structured surveillance approach. While routine endoscopic screening is not currently recommended in breast cancer patients, our findings support the potential value of a risk-adapted strategy, particularly in individuals receiving long-term endocrine therapy or presenting persistent digestive symptoms.

To our knowledge, this study is among the few to combine both colonoscopic and upper gastrointestinal evaluation in patients with hormone receptor-positive breast cancer, providing a more comprehensive assessment of digestive tract involvement in this population.

## 5. Conclusions

This study highlights a possible association between hormone receptor-positive breast cancer and the presence of gastrointestinal lesions, including colorectal polyps and gastric premalignant conditions, detected through endoscopic evaluation. Our findings demonstrate that a considerable proportion of patients in this cohort harbored clinically relevant digestive pathology, even in the absence of prior gastrointestinal screening. However, due to the absence of a control group, no conclusions can be drawn regarding whether the prevalence of these lesions is higher than in the general population or specifically related to hormonal receptor status.

These observations are consistent with previous epidemiological studies reporting associations between breast cancer survivorship and gastrointestinal malignancies, potentially reflecting shared hormonal, metabolic, and lifestyle-related risk factors [25].

While endocrine therapy remains an essential part of treatment and significantly improves survival outcomes, its long-term systemic effects continue to warrant investigation. Aromatase inhibitors, widely used in ER/PR-positive breast cancer, have been associated with metabolic and endocrine alterations that may extend beyond breast tissue [26]. Furthermore, experimental evidence demonstrating aromatase expression within gastric mucosal cells suggests a possible role of estrogen in gastrointestinal mucosal homeostasis [27].

Our findings suggest that gastrointestinal symptoms in patients undergoing breast cancer treatment should be evaluated carefully and not attributed solely to treatment side effects without appropriate clinical assessment. Early recognition of underlying gastrointestinal pathology may contribute to improved symptom management and quality of life.

From a clinical perspective, these findings support consideration of gastrointestinal health within the broader framework of breast cancer survivorship care. A personalized approach incorporating individual risk factors such as metabolic comorbidities, lifestyle habits, and family history may help identify patients who could benefit from further gastrointestinal evaluation [28,29].

Further large-scale prospective controlled studies are needed to better define the relationship between hormone receptor-positive breast cancer, endocrine therapy, and gastrointestinal pathology, as well as to determine whether targeted endoscopic surveillance may provide clinical benefit in selected patient populations.

In conclusion, our study emphasizes the importance of maintaining awareness of gastrointestinal health in patients receiving long-term endocrine therapy for breast cancer while highlighting the need for further controlled investigation in this area.

## Figures and Tables

**Table 1:** Assessment questionnaire.

No.	Question	Yes/No
1	Do you wake up in the middle of the night because of abdominal/epigastric pain?	☐ Yes ☐ No
2	Does your abdomen hurt shortly after eating?	☐ Yes ☐ No
3	Do you experience a painful sensation of hunger?	☐ Yes ☐ No
4	Have you experienced unintentional weight loss?	☐ Yes ☐ No
5	Have you noticed any bowel movement changes after starting hormonal therapy?	☐ Yes ☐ No
6	Are you experiencing constipation or loose stools?	☐ Yes ☐ No
7	Are you experiencing bloating during the day	☐ Yes ☐ No
8	Are you nauseous after you eat?	☐ Yes ☐ No
9	Difficult/painful swallowing?	☐ Yes ☐ No
10	Sensation of incomplete evacuation	☐ Yes ☐ No
11	Mucus or blood in stool?	☐ Yes ☐ No

**Table 2:** Lesion S size, Paris classification, and histopathological classification.

Lesion ID/Patient nr	Localization	Size (mm)	Lesion	Paris Classification (for Polyps)	Histopathological Classification
31	Inferior rectum	7	polyp	Ip	Lipoma
11	Ascending colon	2	tumor	IIb	Serrated polyp
7	Upper rectum	1.3	polyp	0	Hyperplastic polyp
39	Inferior rectum	20	polyp	Ip	Fibroepithelial polyp
44	Transverse colon	8	tumor	N/A	Tubular adenoma
46	Sigmoid colon	10	polyp	Ip	Hyperplastic polyp
51	Sigmoid colon	12	polyp	Is	Lipoma
58	Cecum	8	tumor	1sp	Leiomyoma
66	Superior rectum	9	tumor	N/A	Adenocarcinoma
2	Sigmoid colon	1.2	tumor		Leiomyoma
77	Descending colon	9	polyp	IIa	Serrated polyp
93	Descending colon	1.8	polyp	Is	Serrated polyp

## Data Availability

The original contributions presented in this study are included in the article. Further inquiries can be directed to the corresponding author.
